# Causal relationship between gut microbiota and diabetic neuropathy: a Mendelian randomization and 16S rRNA sequencing analysis

**DOI:** 10.3389/fendo.2025.1632406

**Published:** 2025-09-25

**Authors:** Yingqing Hu, Yanqiu Liang, Youyou Lv, Panwei Mu, Ying Wang, Dingbang Huang, Dezhao Liu

**Affiliations:** ^1^ Department of Anesthesiology, The Fifth Affiliated Hospital of Sun Yat-sen University, Zhuhai, China; ^2^ Department of Endocrinology, The Third Affiliated Hospital of Sun Yat-sen University, Guangzhou, China

**Keywords:** gut microbiota, Mendelian randomization, diabetic peripheral neuropathy, 16S rRNA gene sequencing, diabetes mellitus

## Abstract

**Objectives:**

Evidence suggests a link between gut microbiota and diabetes mellitus, yet the specific role in diabetic peripheral neuropathy (DPN) remains elusive. The study aims to explore the association through Mendelian randomization and 16S rRNA gene sequencing analysis.

**Materials and methods:**

Mendelian randomization (MR) analysis was employed to investigate the causal association between gut microbiota and diabetic neuropathy. Diabetes mellitus (DM) and DPN mice models were developed via high-fat diet (HFD) feeding followed by intraperitoneal streptozotocin (STZ) administration at 30 mg/kg (DM group) or 60 mg/kg (DPN group). The occurrence of diabetic neuropathy was determined by evaluating pain-related behavioral parameters in mice. Additionally, fecal samples from mice and patients with diabetic neuropathy were collected, and 16S rRNA sequencing was performed to analyze the composition of gut microbiota.

**Results:**

Mendelian randomization analysis identified 14 gut microbiota species exhibiting a causal relationship with diabetic neuropathy. In animal studies, diabetic neuropathy mice exhibited decreased mechanical pain thresholds and reduced thermal withdrawal latency. Sequencing analyses further revealed significant alterations in gut microbiota composition in both DPN mice and DPN patients compared to control group.

**Conclusion:**

This study integrates Mendelian randomization analysis with 16S rRNA fecal assessments from animal models and clinical patients, revealing that gut microbiota imbalances may contribute to diabetic neuropathy development and providing novel insights for its prevention and therapeutic strategies.

## 1 Introduction

Diabetes mellitus (DM) is a global epidemic, affecting an estimated 536.6 million people in 2021, with projections indicating a rise to 783.2 million people by2045 (1, 2).Diabetic peripheral neuropathy (DPN), affecting about 50% among diabetic patients (3, 4), manifests as debilitating pain and sensory loss, drastically reducing quality of life and imposing substantial healthcare costs—up to 4.2 times higher than diabetes without DPN (5, 6). Currently, the treatment options for diabetic neuropathy are limited to symptom management through glycemic control and lifestyle adjustments (7, 8). Given the significant impact of diabetic neuropathy on public health and the healthcare system, there is an urgent need for in-depth research into this condition.

Current research highlights the gut microbiota’s critical role in DM pathogenesis through inflammation, immunity, and metabolism (9–11). The gut microbiota, being one of the largest microbial ecosystems within the human body, has been indicated that gut microbiota can influence the onset and progression of diabetes by modulating glucose metabolism and insulin sensitivity (12–14). Recent findings suggest that improving the composition of gut microbiota may have a positive effect on the treatment of diabetes (14, 15). Despite existing research indicating that gut microbiota may affect the development of diabetes through various pathways, research on the relationship between gut microbiota and diabetic neuropathy remains limited.

The brain-gut axis, a dynamic bidirectional communication network linking the gut and the central nervous system (CNS), has emerged as a pivotal pathway potentially mediating the effects of gut dysbiosis on distal organs, including the peripheral nerves (16). Gut microbiota secrete hormones that can indirectly modulate host inflammation levels (17) and pain sensitivity through the vagus nerve pathways (18–21). Additionally, research employing obese mouse models shows that the gut microbiota plays a crucial role in dietary regulation-induced changes in energy balance and glucose metabolism (22).These mechanisms are likely intricately linked to the pathogenesis of diabetic neuropathy. Therefore, elucidating the interactions between gut microbiota and diabetic neuropathy holds significant clinical importance for developing novel therapeutic strategies for this condition.

## 2 Materials and methods

### 2.1 Study design

This study is divided into two parts. Initially, Mendelian randomization analysis was conducted to explore the causal relationship between gut microbiota and diabetic neuropathy. Subsequently, 16S rRNA gene sequencing was performed on fecal samples obtained from diabetic neuropathy patients and mice. The comparative analysis of microbial community structures discerned differential bacterial abundances, which served as external validation for the results of the Mendelian randomization analysis.

SPF-grade male C57BL/6J mice (6–8 weeks of age, body weight 21–27 g) were obtained from the Guangdong Medical Experimental Animal Center (License No. (2019) 05073). Mice were housed in a ventilated facility under controlled environmental conditions: temperature 21 ± 2 °C, humidity 60 ± 10%, a 12-hour light/dark cycle, with ad libitum access to food and water.

### 2.2 Ethics statement

The study was conducted in accordance with the Declaration of Helsinki and was approved by the Medical Ethics Committee of the Third Affiliated Hospital of Sun Yat-sen University (No.2021-02-021-01, ChiCTR2100051493). Written informed consent was obtained from all participants prior to the survey. All animal experiments were approved by the Laboratory Animal Ethics Committee of South China Agricultural University (Approval No. 2021D081).

### 2.3 Induction of diabetic and diabetic neuropathy models

Following a 1-week adaptive acclimation period, mice were randomly allocated to three groups via a random number generator: control group (Con group, n=6), diabetes (DM group, n=10), and diabetic peripheral neuropathy (DPN group, n=10). Following 4 weeks of high-fat diet (HFD) feeding, DPN group received intraperitoneal injections of streptozotocin (STZ, 60 mg/kg) for 5 consecutive days, prepared in sodium citrate buffer (pH = 6.0) (23). DM group were subjected to 4-week HFD feeding followed by intraperitoneal STZ administration at 30 mg/kg for 5 days (24). Control group were fed a standard chow diet for 4 weeks and injected intraperitoneally with an equivalent volume of sodium citrate buffer for 5 consecutive days.

**Figure 1:**
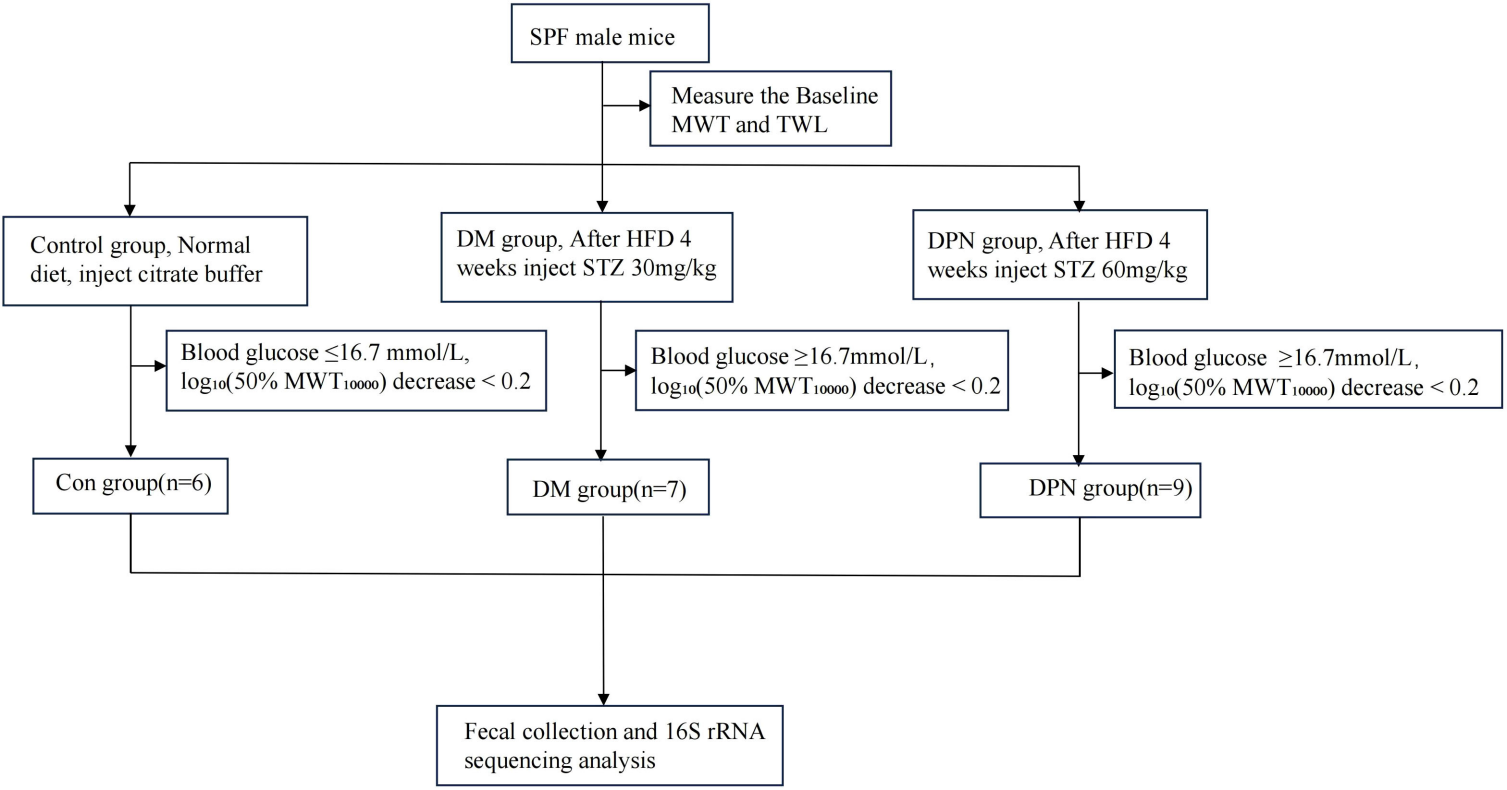
Flowchart of animal experimental procedures.

On days 7, 14, and 21 post-treatment, blood glucose levels were measured via tail vein blood sampling, and behavioral tests were conducted. The endpoint was set at 21 days after treatment, where mice in the DM group with random blood glucose levels ≥16.7mmol/L were selected. For the DPN group, in addition to blood glucose levels, a thermal withdrawal latency (TWL) of less than 20 seconds and log_10_(50%MWT*10000) decrease ≥ 0.2 from baseline were considered indicative of successful model establishment (24–26). The detailed experimental design is elucidated in **Figure 1**.

### 2.4 Behavioral tests

Behavioral assessments included TWL and mechanical withdrawal threshold (MWT) measurements, performed using a hot plate apparatus and Von Frey filaments, respectively.

#### 2.4.1 Thermal withdrawal latency

Mice were placed on a hot plate analgesia meter set at 52 ± 1 °C within a transparent enclosure. TWL was defined as the time from placement to the first occurrence of heat-evoked responses (e.g., paw licking or jumping). Each mouse was tested three times with 10-min intervals, and the mean value was recorded. Mice with TWL >20 seconds were excluded from the study (24).

#### 2.4.2 Mechanical withdrawal threshold

After a 15–30 min acclimation period in a transparent grid-bottomed chamber (until stress behaviors ceased), MWT was measured using “up-down” method with Von Frey filaments (0.4, 0.6, 1.0, 1.4, 2.0 g). Starting with a 1.0 g filament, the stimulus intensity was decreased by one step after three consecutive positive responses or increased after three negatives. Following four consecutive response sequences, the 50% MWT was calculated. To account for Weber’s law, the 50% MWT was multiplied by 10,000 and log_10_-transformed (23, 26).

### 2.5 Data source and participants

Gut microbiota data were sourced from the NHGRI-EBI GWAS Catalog (https://www.ebi.ac.uk/gwas/), specifically selecting 473 microbial taxa ranging from GCST90032172 to GCST90032644 (27). This dataset was generated from a comprehensive genome-wide association study, assessing 7,979,834 human genetic variants in the FINRISK 2002 cohort, comprising 5,959 individuals. The participants in the dataset were aged 24 to 74, with an average age of 49.6, and were drawn from six regions across Finland, comprising 55.1% females and 44.9% males.

The genetic data of DPN patients were obtained from the FinnGen R11 database (https://www.finngen.fi/en). The GWAS dataset comprises 54,913 adult participants, including 3,503 cases and 51,410 controls from a prospective cohort study involving the European population Cases were defined by the presence of ICD-10 codes E10.4, E11.4, E12.4, E13.4, or E14.4. As the initial GWAS data had previously been granted approval by the relevant ethical and institutional review boards, ethical clearance for its use in this study was not required.

As the initial GWAS data had previously been granted approval by the relevant ethical and institutional review boards, ethical clearance for its use in this study was not required.

A total of 38 patients were enrolled between 2021 and 2023 from the Third Affiliated Hospital, Sun Yat-sen University, and allocated into three groups: 13 in the DM group, 15 in the DPN group, and 10 in the Con group. Detailed patient information was retrieved from the management information system and the medical records department of the Third Affiliated Hospital of Sun Yat-sen University. Inclusion criteria for diabetes mellitus were that the patients (i) met the diagnostic criteria for T2DM with random plasma glucose ≥11.1 mmol/L (200 mg/dL), (ii) Fasting plasma glucose (FPG) ≥7.0 mmol/L (126 mg/dL), (iii) 2-hour plasma glucose (2hPG) ≥11.1 mmol/L (200 mg/dL) during an oral glucose tolerance test (OGTT). Inclusion Criteria for Diabetic Neuropathy Patients were that the patients (i). Diagnosis of diabetes mellitus or prediabetes, (ii) Presence of peripheral neuropathy, confirmed by clinical examination and nerve conduction studies, (iii) Exclusion of other causes of neuropathy, including chemotherapy, infections, toxins, etc. Exclusion criteria were that the patients (i)Pregnant or lactating women (ii) Presence of acute diabetic complications (e.g., diabetic ketoacidosis, hyperosmolar hyperglycemic state, severe infection, or acute stress) (iii) Use of antibiotics, prokinetic agents, or prebiotics within 4 weeks prior to fecal sampling (iv) History of gastrointestinal diseases within 4 weeks prior to sampling (v) Gastrointestinal surgery within 6 months prior to enrollment (vi) Concurrent diagnosis of other organic diseases.

### 2.6 Sample collection and 16S rRNA sequencing analysis

Human Sample Collection: Fecal collection tubes were used to collect approximately 10g of fresh fecal matter from the enrolled subjects, focusing on the inner layer of the midsection stool. During collection, urine and toilet bowl walls were avoided. Samples were stored at -80°C within 2 hours and labeled in advance with the patient’s name, gender, age, hospital admission number, and collection time.

Mice Sample Collection: Mice were placed in a clean transparent box lined with sterile filter paper to await defecation. Fecal samples were rapidly collected using sterile cryovials, with 3–5 pellets collected per mouse. In cases of insufficient fecal output or failure to defecate, mice were gently stimulated near the anus with a cotton swab to induce defecation.

Collected samples were immediately frozen in liquid nitrogen and later stored at -80°C.Fecal DNA was extracted using the MagPure Soil DNA LQ Kit (D6356-02, Magen). 100–200 mg of fecal samples were homogenized with 500 mg glass beads in 2-ml tubes containing 0.9 ml Buffer SOL and 90 μl Buffer SDS via bead beating, followed by bacterial lysis at 70 °C for 10 min. After centrifugation at 12,000×g for 1 min, 800 μl of the supernatant was transferred to 1.5-ml tubes, mixed with 150 μl Buffer PS, and centrifuged again at 12,000×g for 5 min. The resulting 450 μl supernatant was subjected to automated purification using the KingFisher Flex platform with the pre-programmed MagPure StoolDNA KF protocol. Extracted DNA was quantified by Qubit dsDNA BR Assay, validated via 1% agarose gel electrophoresis, sealed, and stored at -25 °C.

The V3–V4 hypervariable regions of 16S rDNA were amplified using primers 343F (5’-TACGGRAGGCAGCAG-3’) and 798R (5’-AGGGTATCTAATCCT-3’) through a two-round PCR approach. The first round included a 5-min denaturation at 94 °C, 26 cycles of 94 °C for 30 s, 56 °C for 30 s, 72 °C for 20 s, and a 5-min extension at 72 °C. After purification, the second round was performed with 7 cycles under identical conditions. Final PCR products were purified using Ampure XP beads, eluted, barcoded, and sequenced on an Illumina MiSeq platform with paired-end reads.

Raw paired-end sequencing reads were filtered to obtain clean data, which were then assembled using FLASH software. Subsequently, DADA2 in QIIME2 (2020.11) was employed for denoising, chimeric sequence removal, and amplicon sequence variant (ASV) generation.

### 2.7 Gut microbiota data analysis

For the analysis of gut microbial diversity, both alpha and beta diversity were assessed. Alpha diversity, which reflects species richness within a microbial community, was evaluated using the Chao1 index and the Shannon index. Beta diversity, which represents differences in microbial composition between groups, was analyzed via Principal Coordinates Analysis (PCoA).

Additionally, we analyzed the gut microbiota at the phylum level. The top ten most abundant phyla were selected, and statistical differences between groups were examined using the Kruskal–Wallis rank-sum test. Statistical significance was defined as *P* < 0.05.

### 2.8 Two-sample MR

Eligible genetic instrumental variables (IVs) were selected based on the following criteria: (1) a genome-wide significance threshold of *P* < 5 × 10^-5^, (2) exclusion of SNPs in linkage disequilibrium (LD) with an r² threshold of *P <*0.001 and an LD window set to 10,000 kb; and (3) evaluation of the robustness of IVs through F-statistic analysis, where the F-statistic was calculated using the formula: *F = F=beta^2^/se^2^*, with a threshold of F > 10 (28, 29).

In this study, Mendelian randomization (MR) analyses were performed using multiple approaches to assess causal effects, including inverse-variance weighted (IVW), MR-Egger, weighted median estimator (WME), weighted mode (WM), and simple mode (SM) methods. Given its higher statistical power in simulation studies, the IVW method served as the primary analytical approach. Causal associations were considered statistically significant if they met the following criteria: (1) IVW analysis yielded *P* < 0.05, and (2) the effect direction was consistent across supplementary MR methods (30).

To evaluate the robustness of Mendelian randomization results, potential horizontal pleiotropy was assessed using MR-Egger regression and MR-PRESSO methods (31). Heterogeneity among instrumental variables was examined by applying Cochran’s Q test to the inverse-variance weighted estimates, with *P* < 0.05 suggesting significant heterogeneity (32).

### 2.9 Statistical analysis

All analyses were performed using R software (version 4.4.1). MR analysis was performed using the R-based package “TwoSampleMR” and the “MR-PRESSO” package was used to conduct multiplicity tests.

Statistical analyses were conducted using SPSS (Version 25). For normally distributed quantitative data, results are presented as mean ± standard deviation (SD), with independent sample t-tests used for between-group comparisons. Analysis of variance (ANOVA) was applied for multiple-group comparisons. Non-normally distributed data were described by median and interquartile range (IQR, P25–P75) and analyzed using non-parametric tests (Mann-Whitney U or Kruskal-Wallis test). Spearman,s rank correlation coefficient was used for correlation analysis. Statistical significance was defined as *P* < 0.05.

## 3 Result

### 3.1 Mendelian randomization study of gut microbiota and DPN

**Figure 2:**
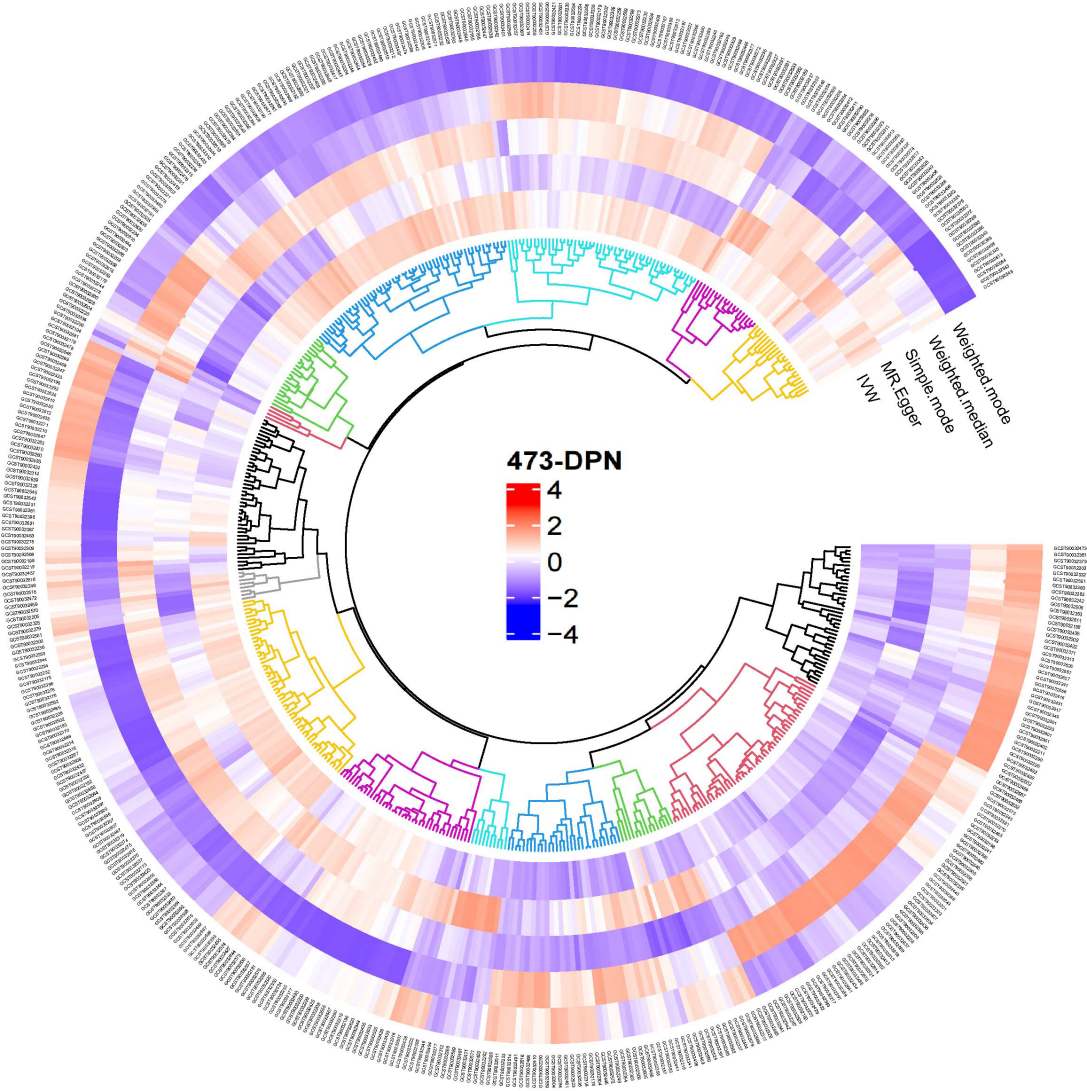
Overview of the causal role of gut microbiota and alopecia areata in MR analysis. Red denotes a positive correlation between gut microbiota and diabetic neuropathy, whereas blue signifies an inverse relationship.

**Figure 3:**
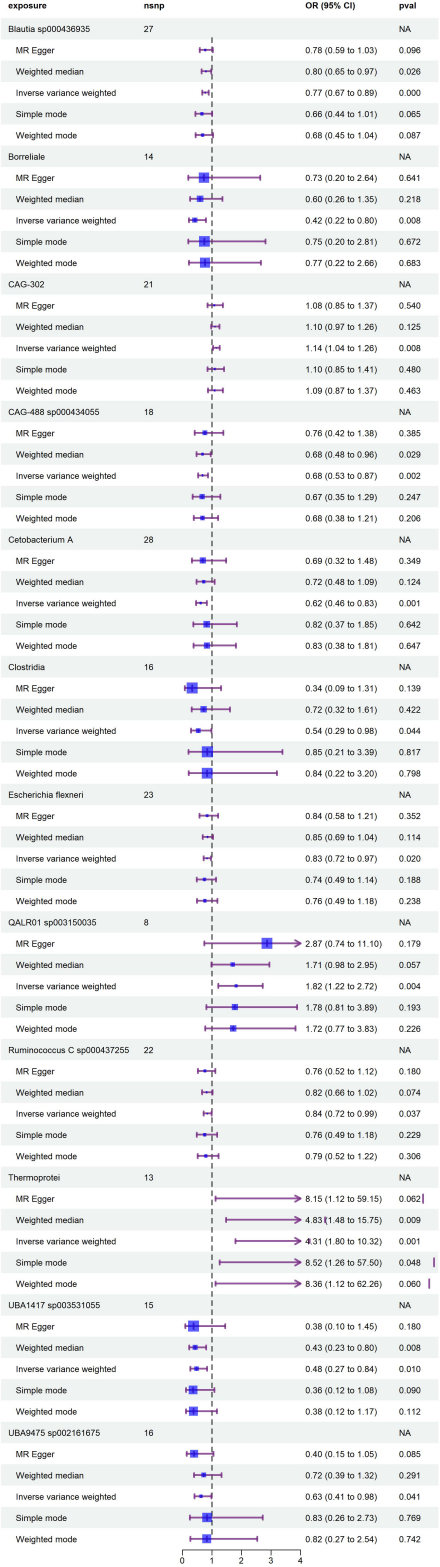
Forest plot illustrates the causal relationship between diabetic neuropathy and gut microbiota.

Following the IV selection criteria outlined in the Methods, 221 SNPs were selected as instrumental variables for gut microbiota. This study analyzed the relationship between 473 gut microbiota and diabetic neuropathy patients, Initially, circular heat maps were employed to visualize all data (**Figure 2**). Subsequent screening identified 14 gut microbiota species exhibiting a causal relationship with diabetic neuropathy, as shown in **Figure 3**. Among them, nine species demonstrated a protective effect against DPN development: *Blautia* sp*000436935* (OR: 0.774, 95%CI: 0.672-0.891, *P* < 0.001), *Borreliale* (OR: 0.422, 95%CI: 0.222-0.801, *P* = 0.008), *CAG-488* sp*000434055* (OR: 0.680, 95%CI: 0.531-0.871, *P* = 0.002), *Cetobacterium A* (OR: 0.620, 95%CI: 0.464-0.830, *P* = 0.001), *Clostridia* (OR: 0.538, 95%CI: 0.294-0.984, *P* = 0.044), *Escherichia flexneri* (OR: 0.835, 95%CI: 0.718-0.972, *P* = 0.020), *Ruminococcus C* sp*000437255* (OR: 0.843, 95%CI: 0.718-0.989, *P* = 0.037), *UBA1417* sp*003531055* (OR: 0.478, 95%CI: 0.272-0.838, *P* = 0.010), *UBA9475* sp*002161675* (OR: 0.633, 95%CI: 0.409-0.982, *P* = 0.041). Conversely, three species were associated with promoting DPN development: *CAG-302* (OR: 1.144, 95%CI: 1.036-1.263, *P* = 0.008), *QALR01* sp*003150035* (OR: 1.820, 95%CI: 1.216-2.723, *P* = 0.004), *Thermoprotei* (OR: 4.308, 95%CI: 1.797-10.324, *P* = 0.001). However, following false discovery rate (FDR) correction, none of these associations retained statistical significance (*P >*0.05, **Supplementary Table S5**).

Leave-one-out sensitivity analysis confirmed the robustness of the initial causal associations, as the overall effect estimates remained consistent after sequentially excluding individual SNPs (**Supplementary Figure S1**). No significant horizontal pleiotropy was detected by the MR-Egger regression intercept test (*P* > 0.05; **Supplementary Table S2**). While Cochran’s Q test suggested the presence of heterogeneity for the gut microbiota feature *UBA1417* sp*003531055* (P = 0.036; **Supplementary Table S3**), subsequent analysis using MR-PRESSO found no significant evidence of heterogeneity (*P* > 0.05; **Supplementary Table S4**).

### 3.2 Comparison of body weight, blood glucose, MWT and TWL in mice

During the experiment, all mice in the Con group maintained blood glucose levels ≤16.7 mmol/L. In the DM group, two mice did not meet the hyperglycemia criterion (blood glucose < 16.7 mmol/L), and one mouse showed a log_10_-transformed 50% mechanical withdrawal threshold (MWT_10000_) decrease greater than 0.2, leading to their exclusion from the study. Similarly, one mouse in the DPN group was excluded due to a log_10_-transformed 50% MWT_10000_ decrease less than 0.2. Consequently, the final sample sizes were 6 mice in the Con group, 7 in the DM group, and 9 in the DPN group.

Baseline comparisons of blood glucose levels and body weights among the three groups showed no significant differences (*P* > 0.05). At the experimental endpoint, as presented in **Table 1**, body weights remained statistically unchanged across groups (p > 0.05). However, significant differences in blood glucose levels were observed (*P* < 0.05). Both the DM and DPN groups exhibited significantly higher blood glucose levels compared to the Con group (p < 0.05), while no significant difference was found between the DM and DPN groups (*P* > 0.05).

**Table 1:** Comparison of baseline and endpoint body weight, blood glucose, MWT and TWL among three groups of mice.

Group	Body weight		Blood glucose levels		50%MWT *10000 (g)		TWL	
	baseline	Endpoint	baseline	Endpoint	baseline	Endpoint	baseline	Endpoint
Con	23.91 ± 1.18	28.86 ± 1.60	9.67 ± 1.05	13.08 ± 1.43	1.25/0.54	1.88/0.92	10.83 ± 2.48	13.34 ± 1.50
DM	24.70 ± 1.80	26.97 ± 2.75	10.03 ± 1.58	22.34 ± 3.88	1.23/0.38	1.30/0.45	11.43 ± 1.63	10.43 ± 1.90
DPN	24.51 ± 1.54	27.64 ± 2.61	9.92 ± 1.91	25.60 ± 4.89	1.25/0.21	0.72/0.18	11.67 ± 1.73	7.23 ± 0.83

As shown in **Table 1**, there were no significant differences in baseline in log10(50%MWT10000) and TWL values among the three groups of mice (*P* > 0.05). At the experimental endpoint, significant intergroup differences were observed in log_10_(50%MWT*10000) and TWL (*P* < 0.05). The log_10_(50%MWT*10000) of the DPN group was significantly lower than that of the Con and DM groups (*P* < 0.05), while no significant difference was found in log_10_(50%MWT*10000) between the Con and DM groups (*P* > 0.05). Thermal nociception tests showed that compared with the Con group, the TWL of both the DM and DPN groups was significantly shortened (*P* < 0.05).

These results collectively demonstrate the successful establishment of both type 2 diabetes mellitus and DPN mice models.

### 3.3 Analysis of intestinal microbial composition of mice

**Figure 4:**
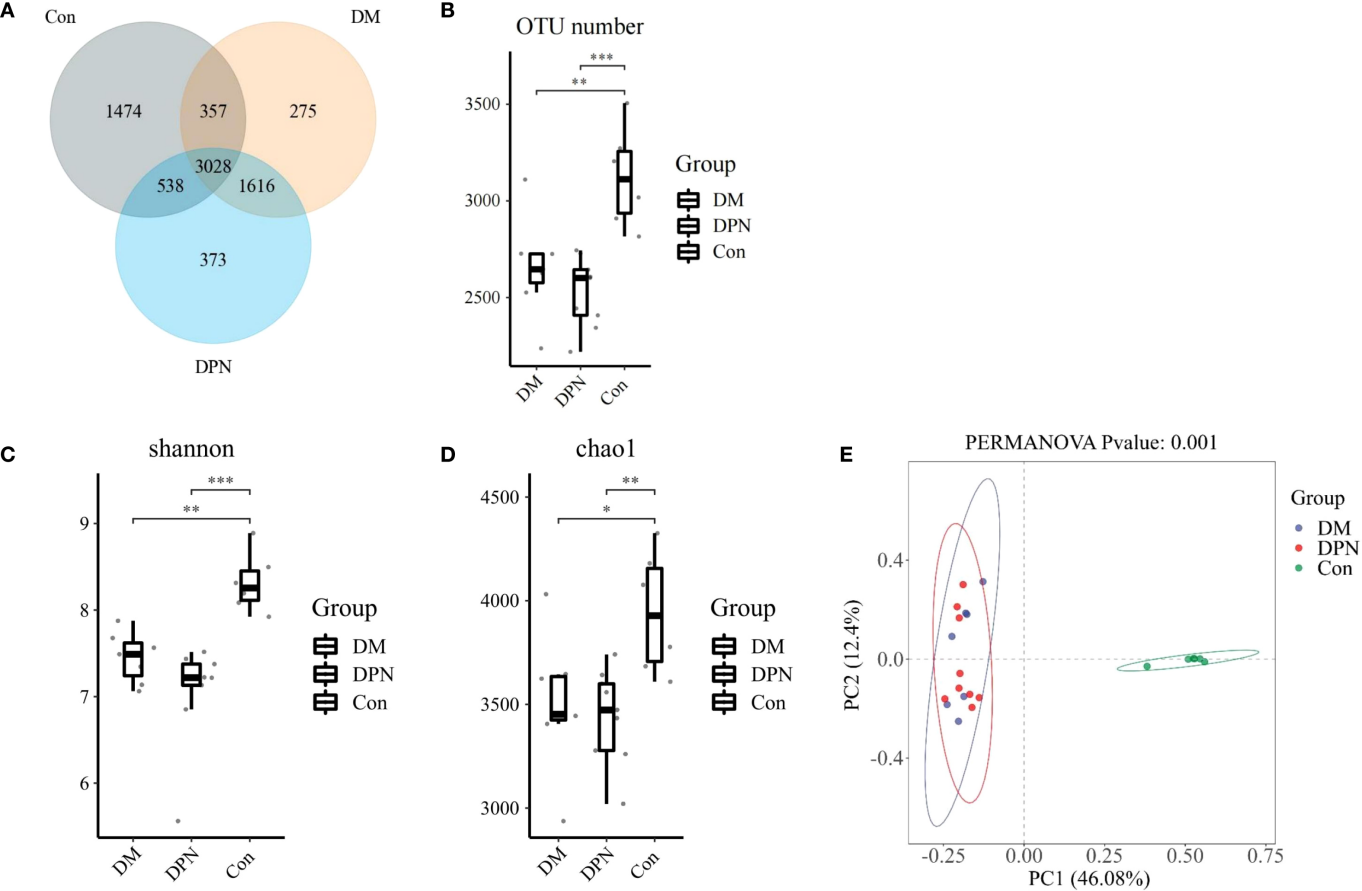
Changes in OTU abundance, alpha diversity, and beta diversity in mice with diabetes and diabetic neuropathy. **(A, B)** Venn diagram and box plot of OTU abundance. **(C, D)** Box plots of alpha diversity (assessed by Chao1 and Shannon indices) across the three groups. **(E)** Principal Coordinates Analysis (PCoA) plot based on Bray-Curtis dissimilarity (beta-diversity) among groups. (*P < 0.05, **P < 0.01, ***P < 0.001).

To investigate alterations in gut microbiota composition associated with diabetes and diabetic neuropathy, fecal samples were analyzed via 16S rRNA gene sequencing. Statistical analysis of operational taxonomic unit (OTU) abundance per mouse revealed that compared with the Con group, the OTU abundance in both DM group and DPN group was significantly reduced (*P* < 0.01 **Figure 4B**). Venn diagram showed that a total of 3,028 OTUs were shared among the three groups; among them, 1474, 275, and 373 unique OTUs were identified in the Con group, DM group, and DPN group, respectively (**Figure 4A**).

To evaluate differences in gut microbial community composition under diabetic and diabetic neuropathic conditions, alpha and beta diversity analyses were performed. In alpha diversity, compared with the Con group, both the DM group and DPN group exhibited a decreasing trend in Chao 1 index and Shannon index, while no statistically significant difference was observed between the DM group and DPN group (**Figures 4C, D**). Beta diversity analysis based on Bray-Curtis distance indicated that there were significant differences in gut microbiota structure among the three groups (**Figure 4E**).

**Figure 5:**
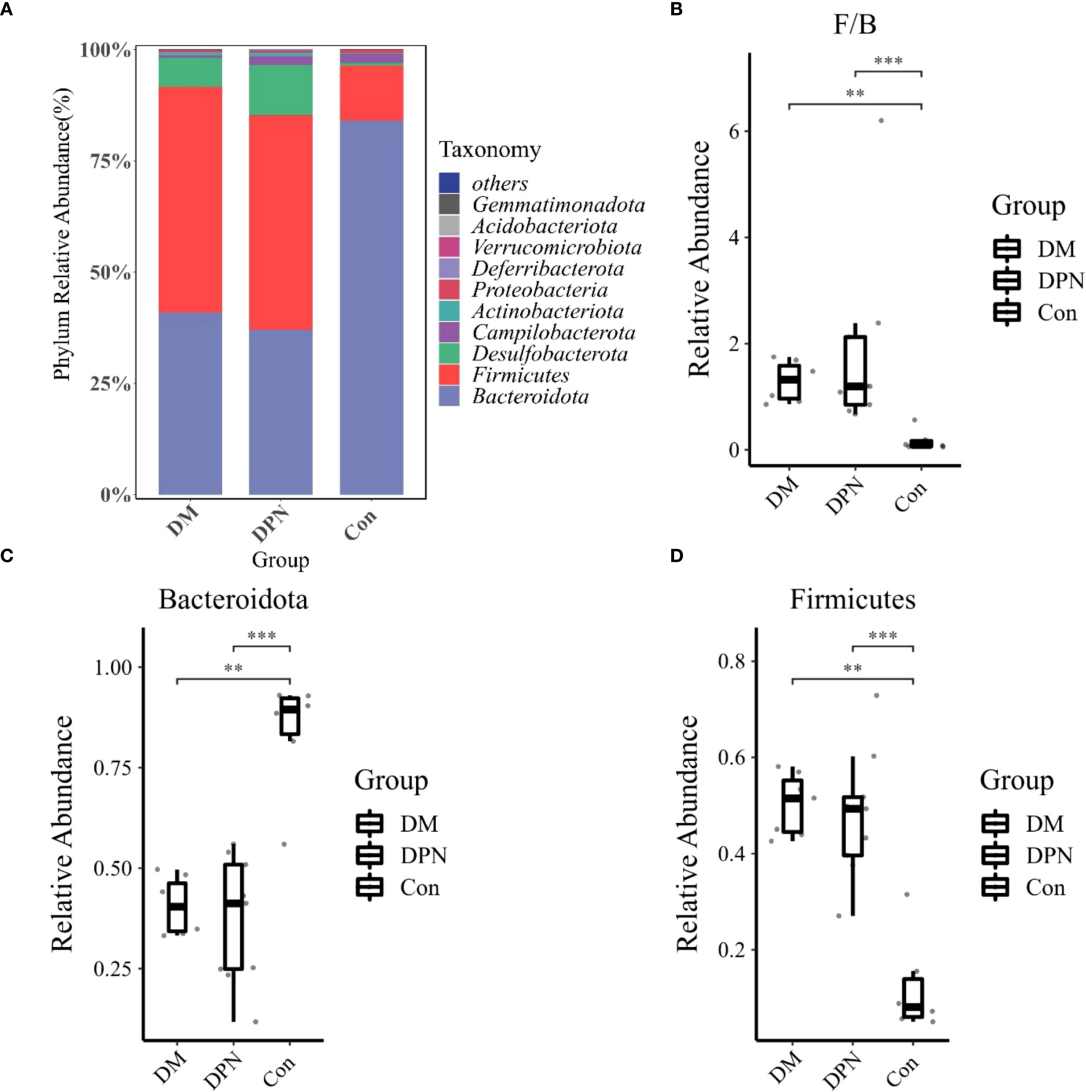
Gut Microbiota Composition at the Phylum Level. **(A)** Bar plot depicting phylum-level microbial abundance structure. **(B)** Box plot of the Firmicutes/Bacteroidetes (F/B) ratio. **(C, D)** Box plots showing the relative abundances of Bacteroidetes and Firmicutes, respectively. (**P < 0.01, ***P < 0.001).

Analysis of the microbial composition at the phylum level indicated that Firmicutes and Bacteroidetes were the dominant phyla in the gut microbiota across all three groups (**Figure 5A**). Relative to the Con group, both the DM and DPN groups displayed an increased relative abundance of Firmicutes and a decreased relative abundance of Bacteroidetes (**Figures 5C, D**). Consequently, the Firmicutes/Bacteroidetes (F/B) ratio was higher in both the DM and DPN groups compared to the Con group (**Figure 5B**).

### 3.4 Clinical sample test results

The baseline data of the study participants are shown in **Table 2**. Subsequent analysis of gut microbiota composition in individuals with diabetes, diabetic neuropathy, and healthy controls revealed no statistically significant differences in OUT abundance among the three groups (*P* > 0.05, **Supplementary Figures S2A, B**). In the α-diversity analysis, compared with the Con group, the Shannon index in both the DM group and DPN group was significantly reduced (*P <*0.05, **Supplementary Figure S2C**), while there was no statistically significant difference in the Chao1 index among the groups(*P* > 0.05, **Supplementary Figure S2D**). Principal Coordinates Analysis (PCoA) based on Bray–Curtis dissimilarity was used to visualize β-diversity, revealing significant differences in gut microbiota composition among the three groups (*P <*0.05, **Supplementary Figure S2E**). Further analysis of the gut microbiota structure in the human cohort at the phylum level revealed that, consistent with the findings in mice. Firmicutes and Bacteroidetes were the dominant phyla across all three human groups (**Supplementary Figure S3A**). However, no statistically significant differences in their relative abundances were observed between groups (**Supplementary Figures S3B-D**).

**Table 2:** Baseline characteristics of the study population.

Characteristic	DPN group (n=15)	DM group (n=13)	Con group (n=10)	P
Male, n (%)	9(60)	7(54)	3(30)	0.320
Female, n (%)	6(40)	6(46)	7(70)	
Age, mean (SD), years	55.05 ± 10.64	55.71 ± 10.33	50.90 ± 10.88	0.529
BMI, mean (SD), kg/m2	24.13 ± 3.21	23.09 ± 3.47	23.32 ± 2.91	0.630
ALT, mean (SD), U/L	16.33 ± 3.75	16.57 ± 4.27	16.50 ± 2.55	0.981
AST, mean (SD), U/L	17.10 ± 6.28	19.21 ± 9.53	15.20 ± 7.24	0.444
LDL, mean (SD), mmol/L	2.80 ± 1.01	22.47 ± 1.11	2.67 ± 0.68	0.633
HDL, mean (SD), mmol/L	0.91 ± 0.22	0.92 ± 0.42	1.11 ± 0.21	0.194
FBG, mean (SD), mmol/L	6.93 ± 2.88	6.74 ± 1.70	4.81 ± 0.70	0.116

## 4 Discussion

A growing body of evidence suggests a correlation between DPN and alterations in the composition and diversity of the gut microbiota (33, 34). This study integrates two-sample MR analysis with 16S rRNA gene sequencing to investigate the potential causal relationship between gut microbiota and DPN. Results revealed gut microbial dysbiosis in patients with DPN, characterized by statistically significant reductions in bacterial alpha diversity and beta diversity, as well as gut microbiota composition.

In this study, we successfully established a model of diabetic neuropathy mice using high-fat diet combined with STZ injections. To evaluate the degree of peripheral nerve injury in the model, behavioral tests —including TWL and MWT measurements—were conducted. Previous studies have highlighted that mechanical allodynia and thermal hyperalgesia are among the most common and detectable symptoms of DPN (35), and these two methods are widely used for efficacy evaluation in STZ-induced DPN mice models. Over three consecutive weeks of behavioral testing following STZ injection, we observed significant decreases in the mechanical withdrawal threshold (MWT) and thermal withdrawal latency (TWL) in the DPN group compared to their pre-modeling baseline values. These results confirm the presence of pain hypersensitivity and confirming the successful establishment of the DPN model.

The brain-gut axis is a complex bidirectional regulatory network that modulates the host’s physiological state through interactions between the gut microbiota and the central nervous system (19). Studies have shown that an imbalance in the gut microbiota can affect the release of inflammatory mediators, thereby stimulating pain receptors involved in the modulation of pain perception (10, 36). In line with this, our microbial diversity analysis revealed significant reductions in both alpha- and beta-diversity in mice and human subjects with diabetic neuropathy, reflecting diminished species richness and substantial structural alterations within the microbial community. These findings are consistent with a state of microbial imbalance, which may further propagate inflammatory responses and exacerbate the progression of neuropathy (37).

In the two-sample Mendelian randomization analysis, we initially identified 14 gut microbial taxa potentially causally linked to diabetic neuropathy. Most of these taxa belong to the phyla *Firmicutes* and *Bacteroidetes*, which together account for more than 90% of the human gut microbiota (38). This study observed an increased abundance of *Firmicutes* and a decreased abundance of *Bacteroidetes* in patients with diabetic neuropathy, which is consistent with findings from previous studies (39, 40). Specifically, compared with the control group, both the DM and DPN mouse groups exhibited a significantly increased *Firmicutes/Bacteroidetes* (F/B) ratio. Evidence suggests that *Firmicutes* abundance is positively correlated with obesity, whereas *Bacteroidetes* shows a negative correlation with obesity (37, 41). Furthermore, several studies have demonstrated that an elevated F/B ratio is implicated in the pathogenesis of various neurological disorders, including neuropathic pain and cognitive impairment (42, 43).

However, analysis of human gut microbiota revealed no statistically significant differences in the abundances of *Firmicutes* and *Bacteroidetes*. This lack of significance could be due to the relatively small sample size, as well as potential confounding effects from oral hypoglycemic agents used by participants (39).

This study has several limitations. First, although the results presented here suggest a potential association between gut microbiota and diabetic neuropathy, the underlying mechanisms remain to be fully elucidated. Second, the Mendelian randomization analysis relied exclusively on genetic data from European ancestry populations; this may introduce population stratification bias.

## 5 Conclusions

This study identifies a link between gut microbiota dysbiosis and diabetic neuropathy (DN), which is characterized by decreased alpha diversity and beta diversity of the gut microbiota, as well as alterations in specific bacterial taxa—including a reduction in *Bacteroidetes* abundance and an increase in *Firmicutes* abundance. These findings highlight the potential of targeting the gut microbiota as a therapeutic strategy for diabetic neuropathy.

## Data Availability

The original contributions presented in the study are included in the article/**Supplementary Material**. Further inquiries can be directed to the corresponding authors.
