# Supplementary figures and images for: Causal relationship between gut microbiota and diabetic neuropathy: a Mendelian randomization and 16S rRNA sequencing analysis
Source: Front Endocrinol (Lausanne). 2025 Sep 25;16:1632406. doi: 10.3389/fendo.2025.1632406 (PMC12507566; doi:10.3389/fendo.2025.1632406)

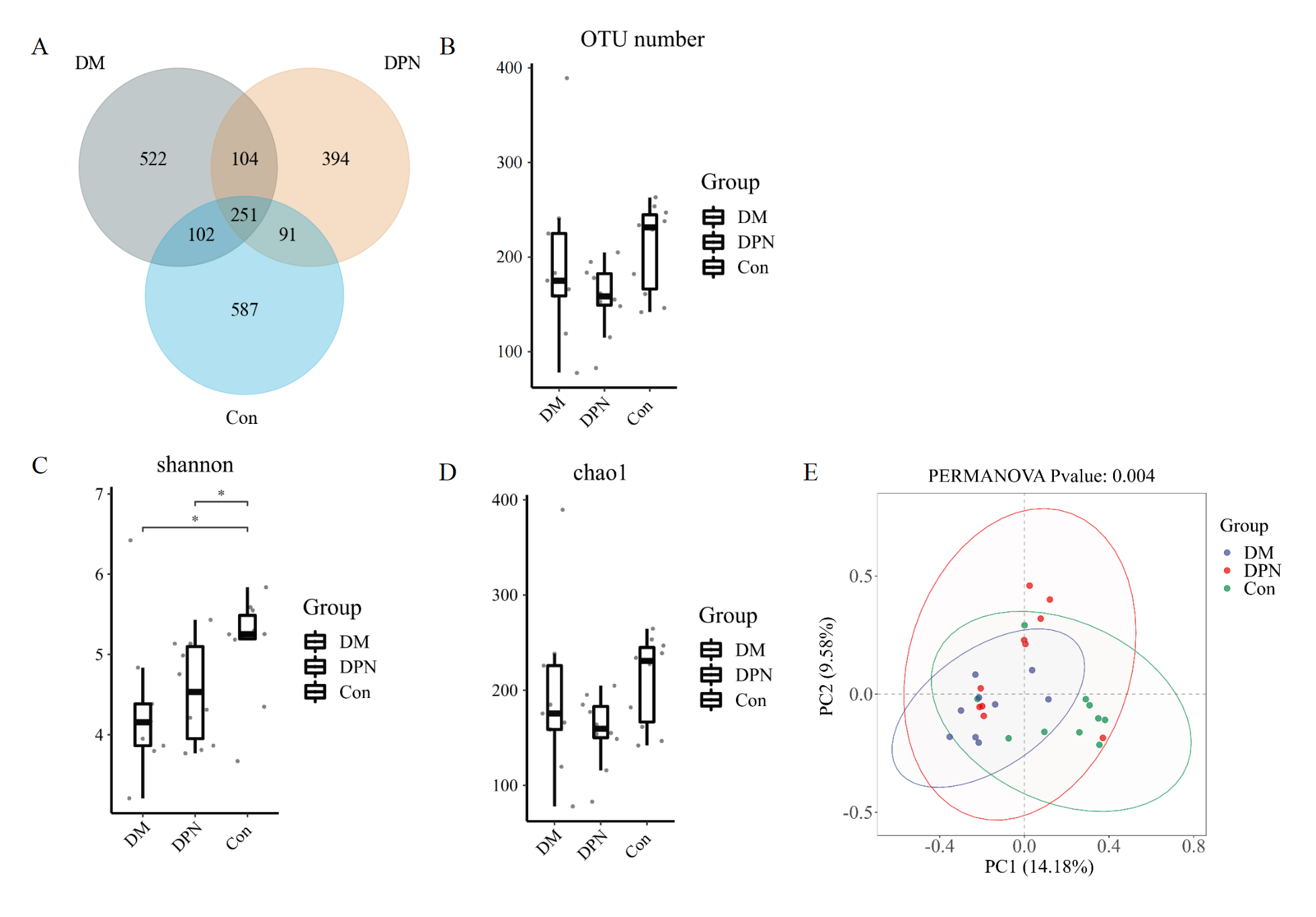

Supplement: Supplementary file 1 [file DataSheet1.zip › supplementary/figure S2.png]

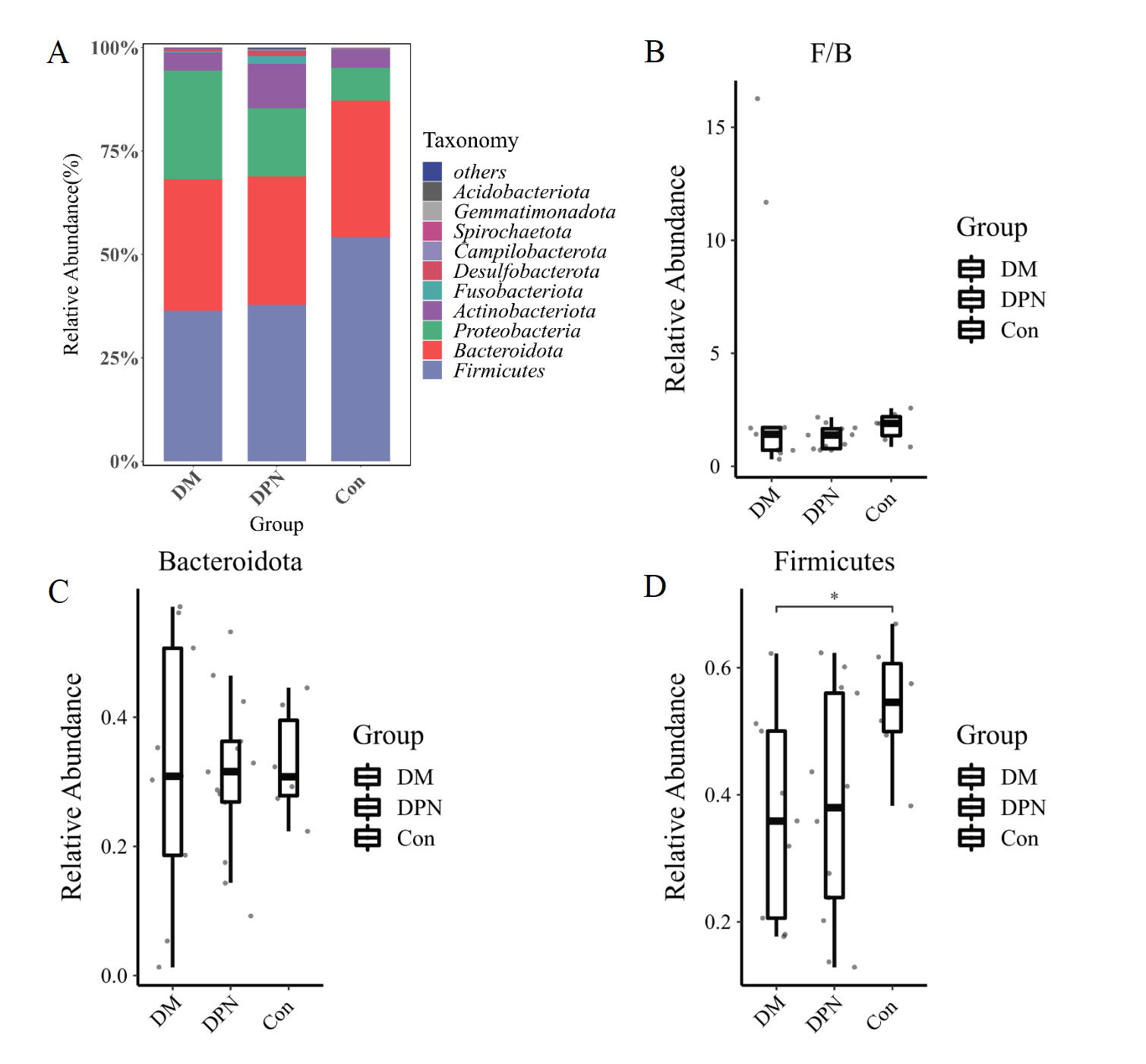

Supplement: Supplementary file 1 [file DataSheet1.zip › supplementary/Figure S3.png]

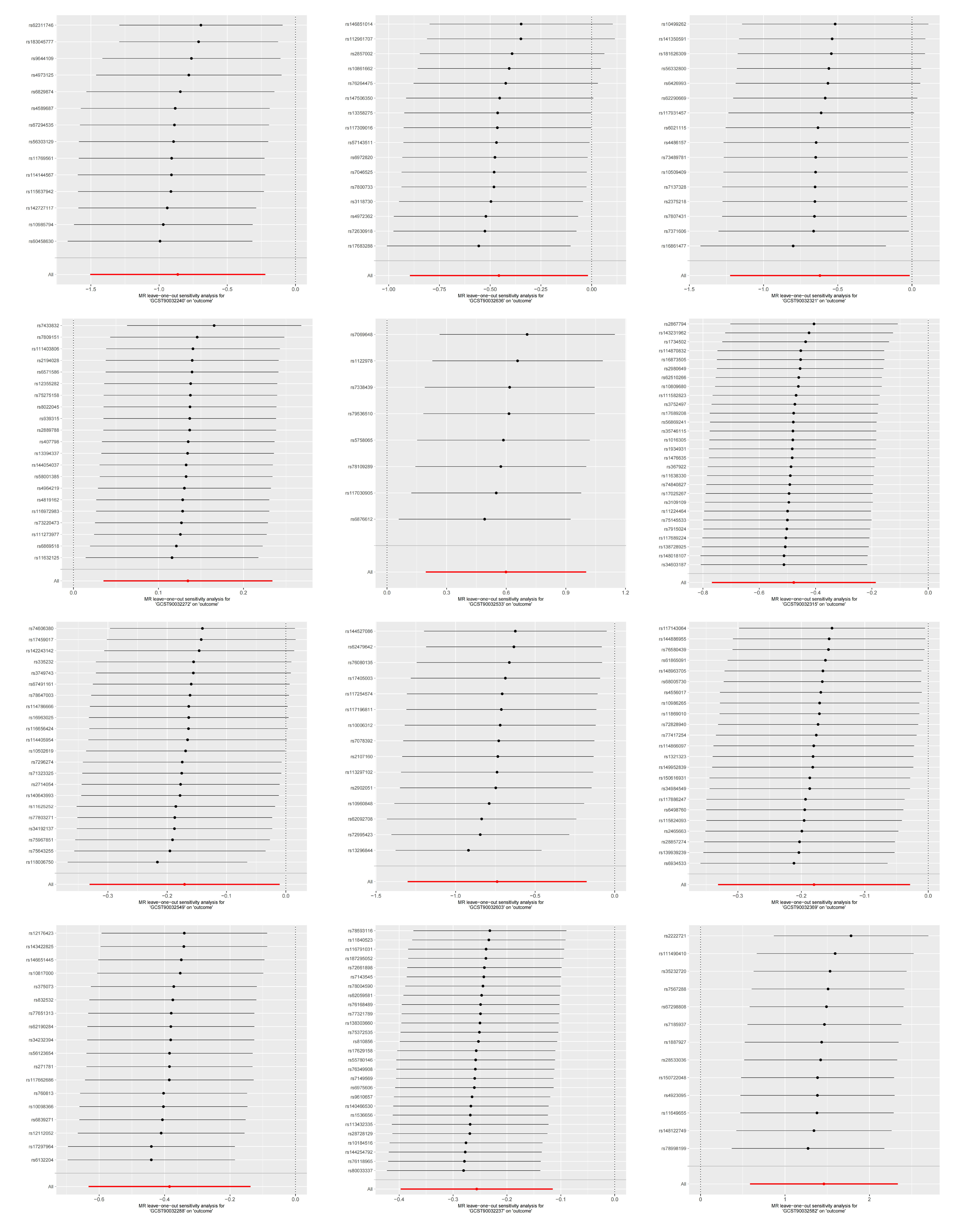

Supplement: Supplementary file 1 [file DataSheet1.zip › supplementary/supplementary figure 1.png]
